# Development of a core genome multilocus sequence typing scheme and life identification number code classification system for Staphylococcus aureus

**DOI:** 10.1099/mgen.0.001486

**Published:** 2025-08-29

**Authors:** Nazreen F. Hadjirin, Iman Yassine, James E. Bray, Martin C. J. Maiden, Keith A. Jolley, Angela B. Brueggemann

**Affiliations:** 1 Nuffield Department of Population Health, University of Oxford, Oxford, UK; 2 Department of Biology, University of Oxford, Oxford, UK

**Keywords:** cgMLST, genotyping, life identification number (LIN) codes

## Abstract

*Staphylococcus aureus* infects both humans and animals, and antimicrobial resistance, including multidrug resistance, complicates the treatment of *S. aureus* infections. Understanding the population structure and distribution of genetic lineages of *S. aureus* is central to understanding the biology, epidemiology and pathogenesis of this organism. This study exploited a large, publicly available dataset of nearly 27,000 *S*. *aureus* genomes to (i) develop a core genome multilocus sequence typing (cgMLST) scheme, (ii) stratify hierarchical clusters based on allelic similarity thresholds, and (iii) define the clusters with a life identification number (LIN) code classification system. The cgMLST scheme characterised allelic variation at 1,716 core gene loci, and 13 classification thresholds were defined, which discriminated *S. aureus* variants across a range of genetic similarity thresholds. LIN code ‘lineages’ and clonal complexes defined by seven-locus multilocus sequence typing were highly concordant, but the LIN codes permitted a wider range of genetic discrimination among *S. aureus* genomes. This *S. aureus* cgMLST scheme and LIN code system is a high-resolution, stable genotyping tool that enables detailed genomic analyses of *S. aureus*.

**Impact Statement**

*Staphylococcus aureus* is a global pathogen that causes a range of invasive and non-invasive diseases, and antimicrobial-resistant strains of *S. aureus* are a major challenge worldwide. Understanding the epidemiology of *S. aureus* is essential to the prevention and treatment of diseases caused by this organism. A new high-resolution, open access core genome multilocus sequence typing (cgMLST) scheme and life identification number (LIN) code system is proposed here, based on analyses of a large, publicly available dataset of nearly 27,000 *S*. *aureus* genomes. This new cgMLST scheme and LIN code system enables detailed analyses of genetic lineages within the *S. aureus* population and can be used to address a variety of questions related to the genomic epidemiology of *S. aureus*.

## Data Summary

All genome sequences and corresponding metadata are available from PubMLST (https://pubmlst.org/organisms/staphylococcus-aureus). Genome accession numbers are available within each isolate record in PubMLST and Data S1.

## Code Availability

The relevant code for data analyses can be found here: https://github.com/brueggemann-lab/pgl_cgmlst_2024.

## Introduction

*Staphylococcus aureus* colonizes healthy humans as well as healthy animals, such as livestock, companion animals and wildlife, but is also associated with a wide range of diseases in both humans and animals. Globally, *S. aureus* caused an estimated 1.1 million human deaths in 2019, and over 100,000 of those deaths were attributable to methicillin-resistant *Staphylococcus aureus* (MRSA) [1]. Antimicrobial resistance (AMR) among *S. aureus* is a major challenge worldwide, and the World Health Organization has declared MRSA, vancomycin-intermediate and vancomycin-resistant *S. aureus* to be a high AMR priority [2].

Bacterial whole-genome sequence data are frequently used to investigate a wide range of questions related to population structure, epidemiology, infection, pathogenesis and evolution. Genome sequencing is increasingly utilized in routine clinical microbiology, public health and surveillance programmes [3]. The seven-locus multilocus sequence typing (MLST) scheme for *S. aureus* has become a standard genotyping methodology and the resulting sequence types (STs) are clustered into clonal complexes (CCs) for analyses [4,5]. The evolution and epidemiology of many of the major *S. aureus* CCs have been investigated in detail since the development of MLST, and these findings have informed the overall understanding of this organism, especially in the context of the emergence and spread of MRSA [6,9].

However, seven-locus MLST has limited resolution, and genotyping tools that analyse more of the available sequence data can provide much more resolution, which has numerous applications. Such approaches include ribosomal multilocus sequence typing (rMLST), whereby genetic variation within the 53 bacterial ribosomal protein genes is characterized, which is especially useful for identifying bacterial species [10]. Core genome multilocus sequence typing (cgMLST) is used to characterize genetic variation among genes that are present in all or nearly all strains of a species, providing a much higher level of genetic resolution, since allelic variation among more than a thousand genes is typically used to define a core genome sequence type (cgST) for each genome. Gene-by-gene cgMLST schemes have been developed for several bacterial species [11,15]. A core genome typing scheme was developed for *S. aureus* in 2014, although new alleles for that scheme can only be submitted for assignment via a commercial platform, and a typing scheme called SaLTy was recently published that characterizes just three core genes [16,17].

More recently, cgMLST schemes developed for *Klebsiella pneumoniae* and *Streptococcus pneumoniae* also included a new life identification number (LIN) barcoding classification system to cluster genomes using a range of similarity thresholds [13,14]. A major advantage of the new LIN code approach is that the resulting barcodes are fixed, and thus, a stable hierarchical nomenclature is defined. Here, a publicly available cgMLST scheme and high-resolution LIN code system are described, which characterizes * S. aureus* variants across multiple levels of genetic resolution, and the high-resolution clusters defined by cgSTs and LIN codes are compared to STs and CCs defined by seven-locus MLST.

## Methods

### Compilation of the PubMLST *S. aureus* genome database and study dataset

A total of 30,487 *S*. *aureus* genome assemblies were available in PubMLST at the time of this study. These had mostly been acquired in two ways (https://pubmlst.org/; accessed on 15 May 2023). First, between 2012 and 2016, all available European Nucleotide Archive (ENA) high-throughput sequencing entries labelled as ‘ILLUMINA’ sequencing and organism as ‘*Staphylococcus aureus*’ (around 25,450 genomes) were assembled using Velvet/VelvetOptimiser and uploaded to the PubMLST *S. aureus* database (https://pubmlst.org/organisms/staphylococcus-aureus) [18,20]. Only minimal metadata were available for these genomes. Second, the remainder of the genomes and provenance data had been submitted by PubMLST database users between 2012 and 2023.

Whole-genome sequences (i.e. closed and draft whole-genome sequences, but not metagenome-assembled genomes) were chosen for inclusion in this study based on the following quality control criteria: (i) total length, 2.6–3.0 Mb (median 2.83±0.2 Mb); (ii) total number of contigs <300; (iii) N50 value >25,000; (iv) G+C content, 32.1–33.1 mol% (consensus range based on published literature); and (v) an rMLST species designation of *S. aureus*. rMLST species designations were performed by analysing each query genome relative to the rMLST genome library, as described previously [14]. Contamination was detected by the presence of multiple alleles at ribosomal loci and/or low rMLST support for *S. aureus*, and contaminated genomes were excluded from any further assessments. In total, 26,677 genomes (330 complete genomes plus 26,347 draft genome assemblies) met the quality control criteria and were included (Data S1, available in the online Supplementary Material). From this dataset, 5,000 genomes were randomly chosen as a development dataset, which included at least one representative of each of the 1,558 unique STs (Data S2 and S3). Randomization was performed with the slice_sample in R (v4.2.3). CCs were defined using the global optimal eBURST algorithm implemented in PHYLOViZ (v2.0.0), and CCs were named after the predicted ‘founder’ sequence type/s [5].

### *S. aureus* core genome loci and allele assignment

Previously, 2,265 core gene loci had been defined in PubMLST based on an early version of the *S. aureus* reference genome MRSA252 (NC_002952.1). These 2,265 loci were re-assessed for inclusion in a new cgMLST scheme, using the latest annotated version of the reference MRSA252 genome (NC_002952.2), to ensure that the cgMLST scheme only included non-paralogous core gene loci that were present in ≥99% of the 26,677 study genomes and core gene loci without repeated sequence issues, to create a robust and reproducible cgMLST scheme (Fig. S1).

Alleles were automatically assigned based on hits to the standard criteria of ≥97% sequence identity and 100% sequence alignment length to exemplar alleles defined for each locus such that all known alleles were within 97% identity of an exemplar of the same length. Core gene loci with missing allele assignments were manually curated and alleles were assigned wherever possible. Loci were excluded from the cgMLST scheme because of sequence quality issues (e.g. truncation, fragmentation, lacking a standard start or stop codon, variability in gene length and multiple allele assignments per assembly) and/or because alleles could not be assigned to at least 99% of the 26,677 study genomes. In total, after automated plus manual curation, 1,716 loci were included in the cgMLST scheme (Fig. S1). Clusters of Orthologous Groups (COG) classifications were predicted using eggNOG-mapper (v2) [21].

### Assignment of cgSTs and definition of LIN codes

A LIN code is a multi-position, integer-based barcode that uses the classification thresholds as ‘bins’ that correspond to cgMLST allelic mismatches. LIN codes for each distinct cgST in the entire dataset of 26,677 genomes were assigned within BIGSdb as described previously: (i) a minimum spanning tree using Prim’s algorithm was constructed, and (ii) based on the order in which novel cgST profiles were encoded in the tree, a LIN code for each distinct cgST was assigned [13,14]. A LIN code is assigned to a cgST by matching it to all currently known LIN-encoded cgSTs to identify its closest match. If the same cgST is found, the same LIN code is assigned, but if not, then a novel LIN code is created. When a new profile is assigned, the bin for the corresponding threshold is incremented [13]. Since universal typing nomenclatures necessitate a central authority, all cgMLST allele, cgST and LIN code assignments should be made via PubMLST, and users submit genomes and corresponding metadata to the PubMLST curation system (https://pubmlst.org/organisms/staphylococcus-aureus). When a new *S. aureus* genome is uploaded to the dataset, the 1,716 core gene loci are scanned and cgMLST alleles are assigned (as described in the section above), followed by the automated assignment of cgSTs and LIN codes.

All genomes that had 25 or fewer missing cgMLST alleles were assigned cgSTs using the BIGSdb automated profile definer (https://github.com/kjolley/BIGSdb/blob/develop/scripts/automation/define_profiles.pl). MSTclust (v0.21.200603ac; default parameters) was used to compute pairwise distances between cgMLST allelic profiles of the genomes in the development dataset (*n*=5,000) and the full dataset (*n*=26,677) [22]. MSTclust was also used to calculate the silhouette (*S*_*t*_) score, a measure of cluster cohesiveness, from the 5,000 genomes using pairwise distances for the full range of values for every pairwise mismatch among the 1,716 loci. The density distribution of pairwise allelic mismatches plus the silhouette score was used to define a set of *S. aureus* classification thresholds using the development dataset of 5,000 genomes. Average nucleotide identity (ANI) was computed using FastANI (v1.33) across all genomes. ANI values were converted to distances using the formula 1-ANI/100. The silhouette score was reassessed using the ANI distance matrix to assess how well genomes grouped within LIN code 4 clusters at the M900 level, using the cluster package (v2.1.8.1) in R.

The concordance between cgST clusters vs. other *S. aureus* molecular typing methods [CC, ST, ribosomal sequence type (rST), SaLTy (v1.0.7) and PopPUNK (v2.6)] [23] was tested by calculating the adjusted Rand Index (ARI) for each classification threshold using the development dataset of 5,000 genomes. The analysis was then repeated using the full dataset to test the concordance between cgMLST and CC, ST and rST [24].

### Phylogenetic analyses

A subset of 1,558 genomes, representing one example of each unique ST, was used to construct a maximum likelihood phylogenetic tree with the 1,716 concatenated core gene sequences, to test the congruence of the phylogenetic tree topology relative to each of the defined LIN code thresholds. Allele sequences for the 1,716 cgMLST loci in each genome were retrieved from the BIGSdb sequence definition database, aligned using MAFFT (v7.515) (missing alleles were treated as gaps) and concatenated [25]. snp-sites (v2.5.1) was used to calculate the number of variant sites in the sequence alignments [26]. Maximum likelihood phylogenetic trees were reconstructed with IQ-TREE (v2.0.3), and the built-in model testing (-m MFP) with 1,000 ultrafast bootstraps (-bb 1,000) was used to determine the best phylogenetic model [27]. Trees were midpoint rooted and visualized in ggtree or iTOL [28,29]. Minimum spanning trees were constructed using GrapeTree and the allelic profiles of the 1,716 core genes [30].

## Results

### Description of the *S. aureus* genome dataset

The country of origin and year of isolation were available in PubMLST for approximately half of the genomes (54.4% and 48.4%, respectively), indicating that *S. aureus* from 72 countries and 6 continents were represented, and they were recovered between 1930 and 2023 (Data S4). Around two-thirds of the 14,510 *S*. *aureus* with a known country of origin were either from the USA (45.8%, *n*=6,645) or the UK (16.7%, *n*=2,420). The source of the *S. aureus* isolate was available for only 11.9% (*n*=3,172) of the genome dataset, of which 1,886 were from humans and 1,120 were from animals (Data S4). In total, 5,440 unique rSTs and 1,558 unique STs were represented, and the 1,558 STs clustered into 90 CCs consisting of 2 to 6,405 members per CC. Collectively, 20 CCs represented 95.6% (*n*=25,510) of all genomes in the dataset (Table 1 and Data S4).

**Table 1.** Twenty CCs represented more than 95% of the genomes in the *S. aureus* dataset

CC	Genome (*n*)	% of dataset
5	6,405	24.0
8	5,547	20.8
22	5,107	19.1
398	1,700	6.4
30	1,514	5.7
1	903	3.4
45	683	2.6
130	549	2.1
15	530	2.0
93	469	1.8
97	384	1.4
133	313	1.2
9	277	1.0
59	243	0.9
188	211	0.8
425	167	0.6
88	143	0.5
121	134	0.5
25	126	0.5
72	105	0.4
Total*	**25,510**	**95.6**

*There were an additional 70 CCs that represented 2–97 genomes each, plus 101 singletons (unclustered STs of 1 genome each) (full list in Table S1).

### The cgMLST genotyping scheme

The 1,716 loci included in the cgMLST scheme were distributed across the *S. aureus* genome, and 72.7% (*n*=1,248) were predicted to encode proteins with COG classified cellular pathways (Fig. S2 and Data S5). Overall, 98.8% (*n*=26,347) of the 26,677 *S*. *aureus* genomes had 25 or fewer missing cgST alleles and thus were assigned a cgST. There were 23,136 unique cgSTs (Data S6).

Among genomes with 25 or fewer missing cgST alleles, the mean number of missing alleles was comparable across CCs, suggesting little phylogenetic bias (Fig. S3). Among the 330 genomes that had more than 25 missing alleles, the median was 37 (range 26–138) missing alleles, but these were not assigned a cgST and LIN code to prevent isolates from appearing to be artefactually closer to each other than they are, due to missing loci being ignored in pairwise comparisons.

### Determination of *S. aureus* classification thresholds

**Fig. 1.**
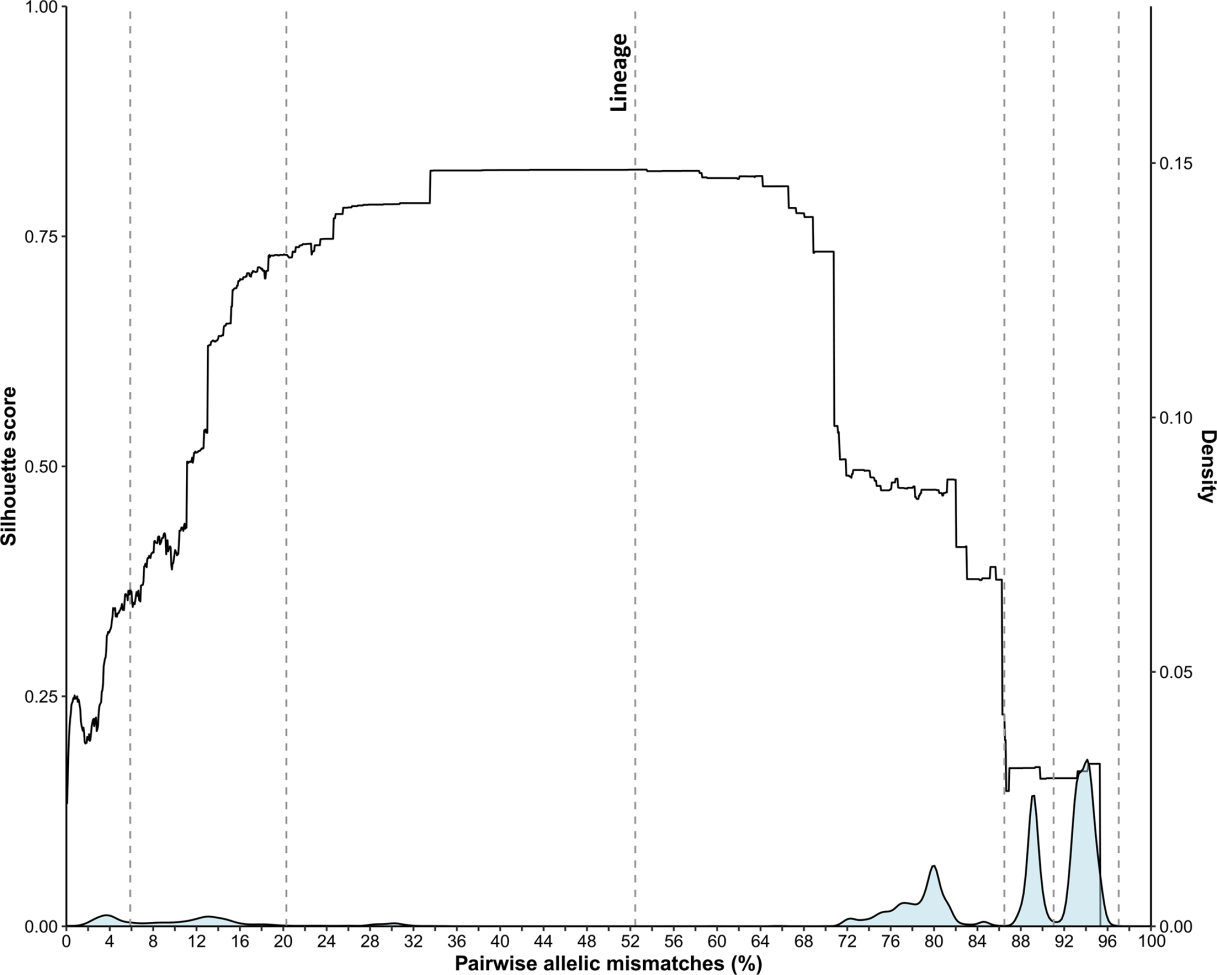
Determination of cgMLST classification thresholds to define the population structure of *S. aureus*. The development dataset of 5,000 genomes, which included at least one example of each of 1,558 unique seven-locus STs, was used in these analyses. Vertical lines mark the first six LIN code classification thresholds, and the lineage designation (LIN code 4, 900 pairwise mismatches) is labelled. Note that the final seven classification thresholds on the left are positioned very closely together and thus not illustrated here.

The development dataset of 5,000 genomes was used to define thresholds of genetic similarity to delineate the *S. aureus* population structure (Fig. 1). A multimodal distribution of pairwise allelic mismatches among the 1,716 cgMLST loci was observed, which included three major peaks centred at 94%, 89% and 80%, and minor peaks around 30%, 13% and 4% allelic mismatches.

A total of 13 thresholds were chosen to provide a range of discrimination levels (Fig. 1). The first threshold was defined at 1,665 pairwise allelic mismatches (i.e. 97.0% genetic dissimilarity): this was the maximum number of core gene allelic differences among all 5,000 genomes. The second threshold was set at 1,562 (91.0%) allelic mismatches to split two major peaks in the distribution. A third threshold was set at 1,484 (86.5%) allelic mismatches to flank the second major peak and coincide with the increasing silhouette score. A ‘lineage’ threshold was set at the highest silhouette score (*S*_*t*_=0.82) and defined genomes with 900 (52.4%) pairwise allelic mismatches. Near the minor peaks, a fifth threshold was set at 348 (20.3%) allelic mismatches, and a sixth threshold was defined at 101 (5.9%) allelic mismatches. Finally, high-level discrimination thresholds were set at 27 (1.6%), 14 (0.8%), 7 (0.4%), 4 (0.2%), 2 (0.1%), 1 (0.06%) and 0 (0%) pairwise allelic mismatches. To further validate this clustering level, we independently computed silhouette scores using pairwise ANI distances and found that LIN code clusters at the lineage level (900 allelic mismatches) were also well-supported based on ANI similarity (*S*_*t*_=0.82) (Figs S4 and S5, Table S2 and Data S7).

### Comparison of the cgST-LIN code scheme with other *S. aureus* genotyping methods

**Fig. 2.**
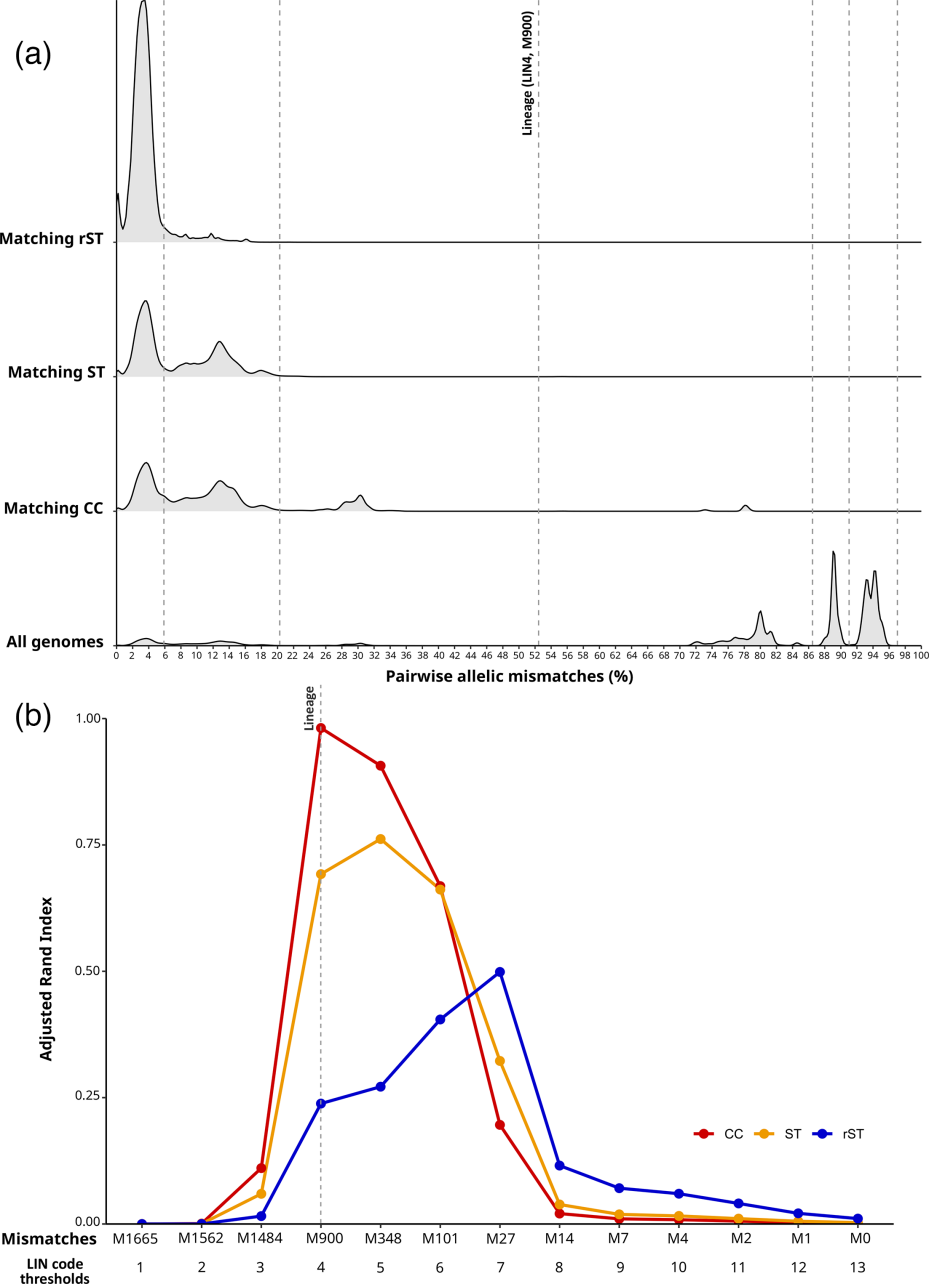
Characterization of *S. aureus* genomes by cgMLST and comparison to other genotyping methods. The results of the analysis of 26,347 *S*. *aureus* genomes with an assigned cgST are illustrated here. (a) The density distributions of pairwise allelic mismatches between genomes belonging to a matching rST, ST or CC taxonomic group. (b) The concordance between clusters of *S. aureus* at each of the 13 LIN code thresholds and the number of pairwise mismatches and the corresponding rST, ST and CC designations. The fourth threshold at 900 pairwise mismatches was designed as a LIN code lineage. M, mismatches.

Expanding the analyses to the dataset of 26,347 genomes with a cgST, the distributions of pairwise allelic differences between genomes belonging to a matching rST, ST or CC group were assessed. The majority of genomes that had matching STs or CCs had fewer than 30% allelic mismatches among the cgST loci, although there was a small group of genomes within CC1, CC5 and CC188 that had matching CCs but around 75–80% allelic mismatches at core gene loci (Fig. 2a).

The concordance between clusters of *S. aureus* at each of the LIN code thresholds and the corresponding rST, ST and CC designations was calculated using the ARI. There was nearly perfect concordance (ARI=0.98) between the LIN code ‘lineage’ (900 pairwise mismatches) and CC; high concordance (ARI=0.76) between LIN code threshold 5 (348 pairwise mismatches) and ST; and moderate concordance (ARI=0.50) between LIN code threshold 7 (27 pairwise mismatches) and rST (Fig. 2b). For comparison, similar analyses that included SaLTy and PopPUNK were run on the development dataset of 5,000 genomes (Fig. S5).

### Increased resolution of *S. aureus* population structure using cgMLST and LIN codes

**Fig. 3.**
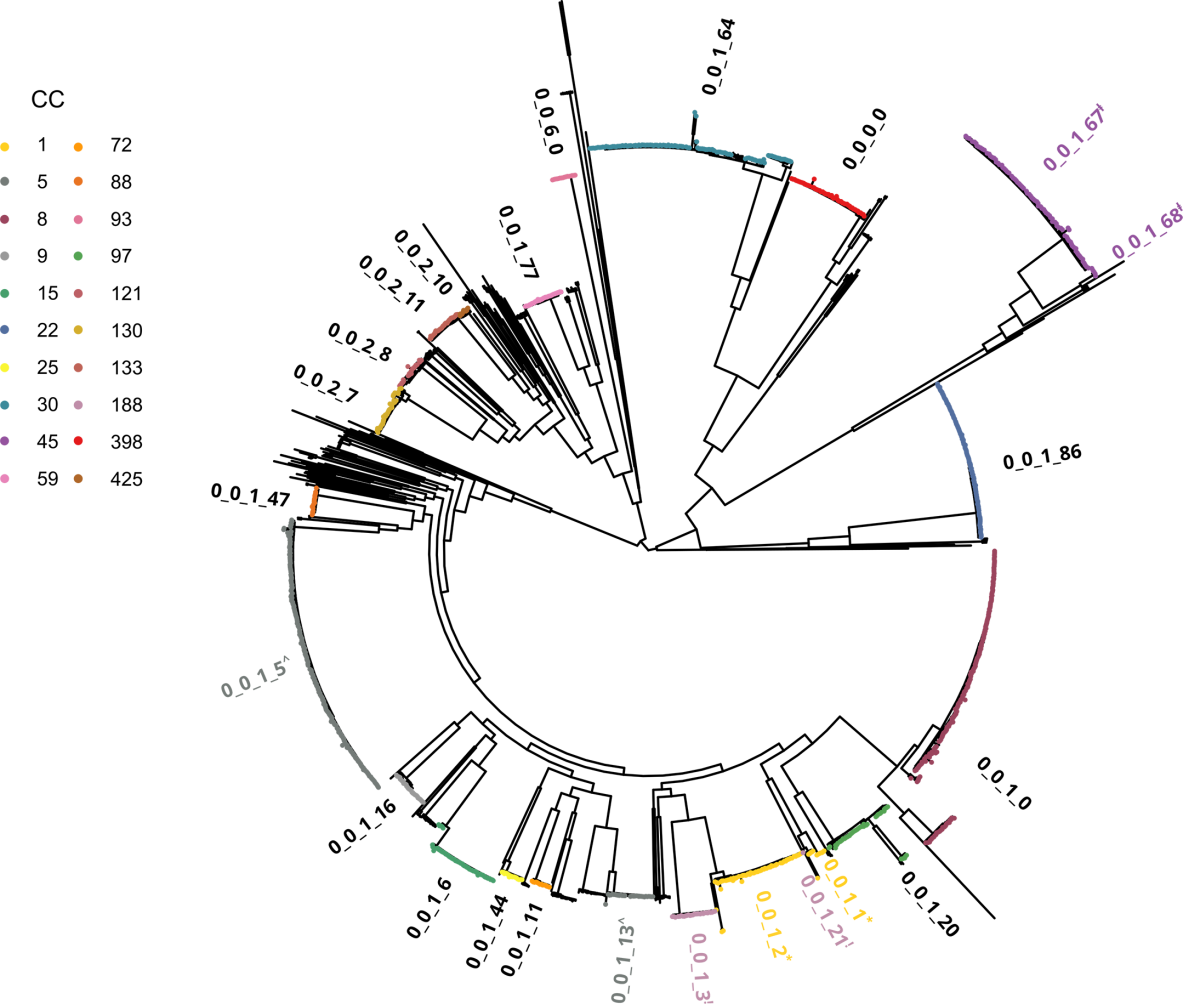
Maximum likelihood phylogeny showing the population structure of *S. aureus*. The tree was constructed with a concatenated alignment of 1,716 core genome loci from one randomly selected genome representative for each of the 1,558 unique seven-locus sequence types. IQ-TREE was used with the GTR+G model of nucleotide substitution and 1,000 bootstrap replicates. The tree is rooted at the midpoint. The 20 most common CCs in the dataset are coloured and annotated with the corresponding LIN code classification threshold 4 lineage designation. Note that CCs 1 (*), 5 (^), 45 (‡) and 188 (!) were split into two LIN code lineages each (see the main text, Table 2 and Figs4  5).

A maximum likelihood phylogenetic tree was constructed using concatenated nucleotide sequence alignments of the 1,716 cgMLST loci of one randomly chosen genome representative for each of the 1,558 unique STs, and the tree was annotated with the 20 most common CCs in this dataset and their corresponding LIN code lineages. This analysis demonstrated concordance between CC and LIN code lineage (Figs 3 and S6).

**Table 2.** Twenty-two LIN code lineages represented more than 95% of the genomes in the *S. aureus* dataset

LIN code lineage	Genomes in lineage (*n*)	% of dataset*	Major CC	No. (%) of genomes in the lineage that are also in the major CC
0_0_1_5	6,298	23.9	CC5	6,231 (98.9)
0_0_1_0	5,539	21.0	CC8	5,516 (99.6)
0_0_1_86	5,083	19.3	CC22	5,080 (99.9)
0_0_0_0	1,680	6.4	CC398	1,679 (99.9)
0_0_1_64	1,557	5.9	CC30	1,504 (96.6)
0_0_1_67	637	2.4	CC45	633 (99.4)
0_0_2_7	557	2.1	CC130	546 (98.0)
0_0_1_6	518	2.0	CC15	518 (100)
0_0_1_2	511	1.9	CC1	501 (98.0)
0_0_6_0	467	1.8	CC93	467 (100)
0_0_1_1	398	1.5	CC1	397 (99.7)
0_0_1_20	337	1.3	CC97	332 (98.5)
0_0_2_11	312	1.2	CC133	312 (100)
0_0_1_16	270	1.0	CC9	270 (100)
0_0_1_77	240	0.9	CC59	238 (99.2)
0_0_1_3	201	0.8	CC188	200 (99.5)
0_0_2_10	164	0.6	CC425	164 (100)
0_0_2_8	149	0.6	CC121	131 (87.9)
0_0_1_47	145	0.6	CC88	142 (97.9)
0_0_1_13	125	0.5	CC5	122 (97.6)
0_0_1_44	121	0.5	CC25	120 (99.2)
0_0_1_11	102	0.4	CC72	102 (100)
Total*	**25,411**	**96.4**	**--**	**--**

*Note that there were 26,347 genomes with an assigned LIN code in the study dataset.

**Fig. 4.**
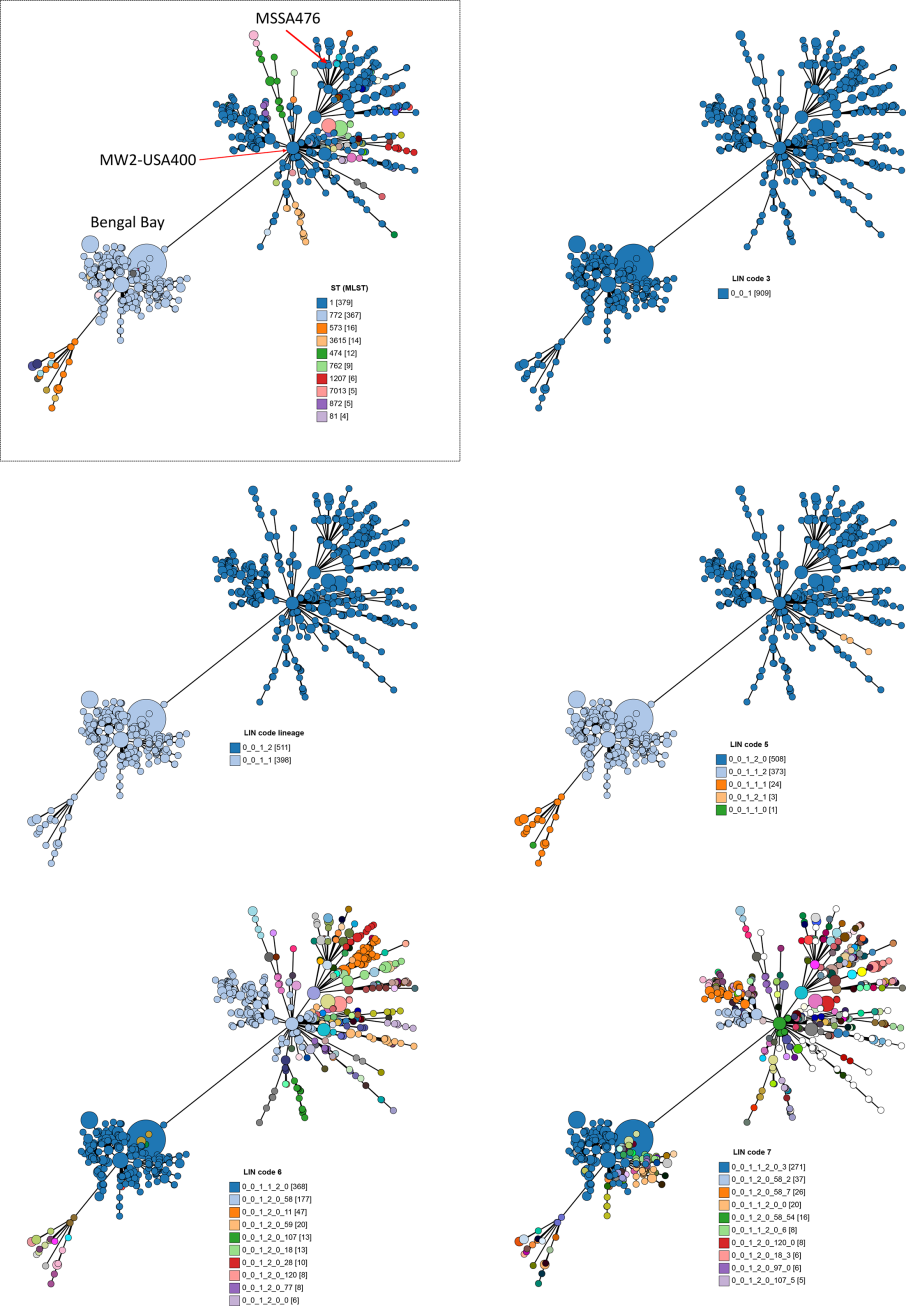
*S. aureus* members of CC1 were split into two LIN code lineages. Relationships between the two LIN code lineages 0_0_1_2 (*n* = 511) and 0_0_1_1 (*n* = 398) were visualized with GrapeTree, which plots allelic profiles of the cgMLST loci. The same minimum spanning tree is shown in each panel, coloured by ST or LIN code. The *S. aureus* in the tree in the boxed panel was coloured by seven-locus ST, and the remaining panels were coloured by increasing LIN code classification thresholds. In the legends, the numbers in square brackets indicate the number of *S. aureus* with that ST or LIN code. For illustrative purposes, the legend only shows the 10 most common LIN codes annotated in the LIN code 6 and LIN code 7 trees. Three *S. aureus* from the published literature are annotated as visual examples of the increased discrimination with LIN codes: Bengal Bay lineage (light blue cluster) [33], reference strain MSSA476 [34] and reference strain USA400 [35].

**Fig. 5.**
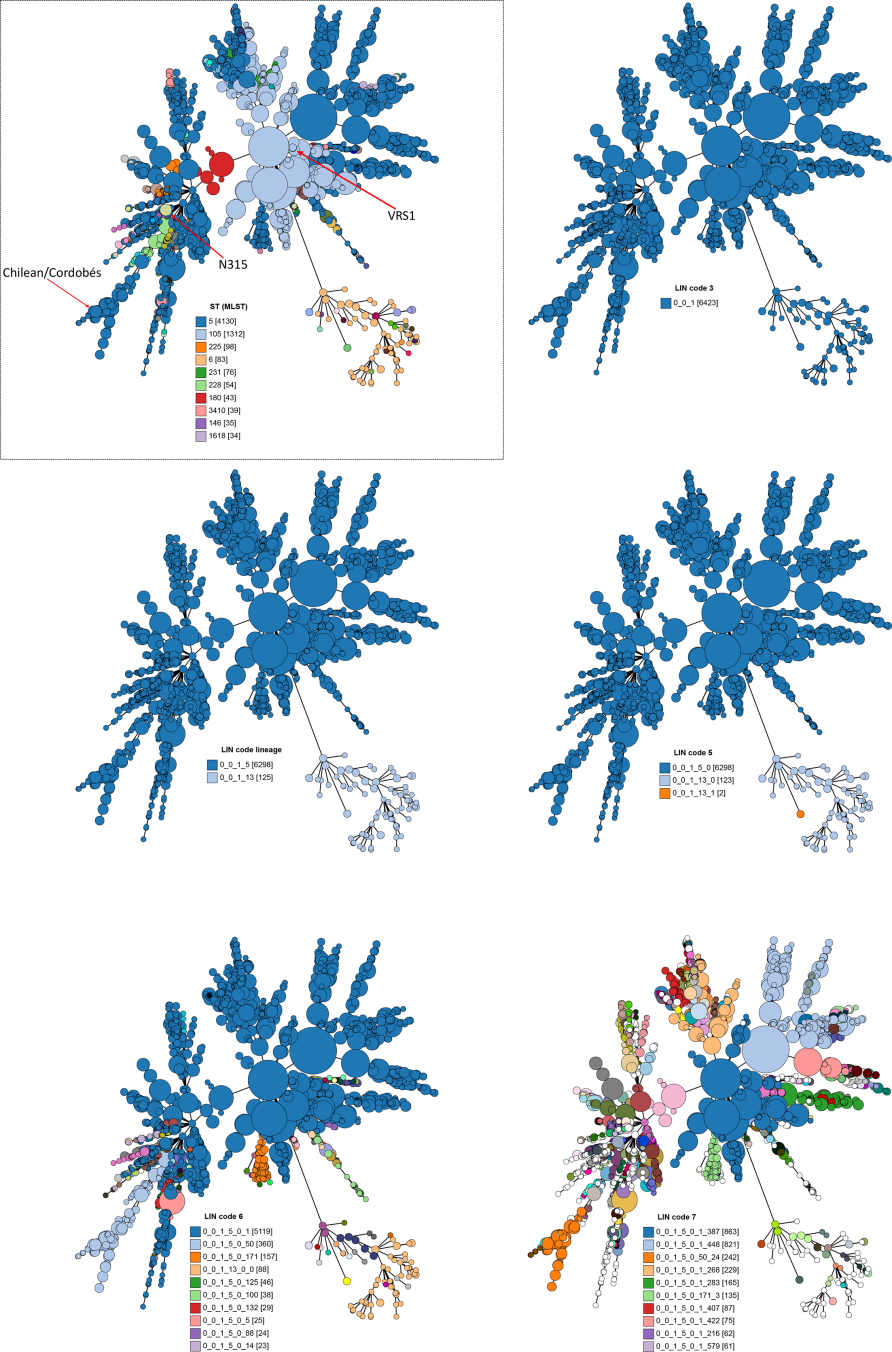
*S. aureus* members of CC5 were split into two LIN code lineages. Relationships between the two LIN code lineages 0_0_1_5 (*n* = 6,298) and 0_0_1_13 (*n* = 125) were visualized with GrapeTree, which plots allelic profiles of the cgMLST loci. The same minimum spanning tree is shown in each panel, coloured by ST or LIN code. The *S. aureus* in the tree in the boxed panel was coloured by seven-locus ST, and the remaining panels were coloured by increasing LIN code classification thresholds. In the legends, the numbers in square brackets indicate the number of *S. aureus* with that ST or LIN code. For illustrative purposes, the legend only shows the 10 most common LIN codes annotated in the LIN code 6 and LIN code 7 trees. Three *S. aureus* from the published literature are annotated as visual examples of the increased discrimination with LIN codes: Chilean/Cordobés lineage [36], reference strain N315 [37] and reference strain VRS1 [38].

Among the 26,347 genomes with an assigned cgST and LIN code, there were 18,455 unique LIN codes. There were 10 LIN code 3 thresholds (8 of which represented more than 1 genome; range 3 to 22,727), 119 LIN code lineages (74 of which represented more than 1 genome; range 2 to 6,298) and 167 LIN code 5 thresholds (96 of which represented more than 1 genome; range, 2 to 6,298) (Data S6).

Twenty-two LIN code lineages comprised 96.4% (*n*=25,411) of the 26,347 *S*. *aureus* genomes with LIN codes. These 22 LIN code lineages also represented the 20 most common CCs in the dataset; however, CC5 *S. aureus* were represented by two LIN code lineages 0_0_1_5 (*n*=6,298 genomes) and 0_0_1_13 (*n*=125 genomes), and CC1 *S. aureus* were roughly evenly split into two LIN code lineages 0_0_1_2 (*n*=511 genomes) and 0_0_1_1 (*n*=398 genomes), respectively (Table 2). Two additional CCs were also split into two LIN code lineages, but only one of each LIN code was represented in >95% of the full dataset: CC45 (0_0_1_67, *n*=637 genomes; 0_0_1_68, *n*=46 genomes) and CC188 (0_0_1_3, *n*=201 genomes; 0_0_1_21, *n*=1 genome) (Table 2 and Data S6).

The relationships among the CC5 and CC1 pairs of LIN code lineages were visualized with GrapeTree, which used the allelic profiles of the 1,716 core gene loci as input data and was useful for demonstrating genetic relatedness among clusters of genomes (Figs4  5). Within each pair of LIN code lineages (900 pairwise mismatches), the two clusters of *S. aureus* were distinct despite being in the same CC. The increased genetic resolution provided by LIN codes was further visualised by annotating the tree at increasing LIN code classification thresholds: LIN code 5 (348 mismatches), LIN code 6 (101 mismatches) and LIN code 7 (27 mismatches). One could further discriminate down to LIN code 12 (one pairwise mismatch; not shown) if desired.

## Discussion

Robust genotyping tools for bacterial pathogens are essential for microbiological research and public health surveillance, and a well-defined, internationally recognized nomenclature is critical for sharing and communicating the genotyping results within the scientific community [31]. The seven-locus MLST scheme has proven to be a robust and widely used typing tool for defining STs and CCs of *S. aureus*, because *S. aureus* has relatively low rates of recombination and the CCs tend to be stable [7,32]; however, whole-genome sequencing enables the characterization of bacterial variants at much greater resolution than seven-locus MLST. This new scheme is an expansion of the seven-locus MLST and CC genotyping and clustering methods, and core gene allelic variation between *S. aureus* strains or within clusters can subsequently be explored further by extracting the relevant genes and gene regions. The recently proposed SaLTy method uses a minimal set of three core genes to characterize high-level epidemiology of *S. aureus* with reasonable success, although SaLTy is not a prospectively curated nomenclature [17]. An ANI-based clustering method has also been described and showed strong concordance with our defined lineage threshold (LIN 4), further supporting its biological relevance [33].

The open access cgMLST scheme and LIN code system described here provide a genotyping scheme for *S. aureus* that precisely characterizes allelic variation among 1,716 core genes. Genomes submitted to PubMLST are curated by a human curator and assigned STs, rSTs, cgSTs and LIN codes. Moreover, accessory genes (which include mobile genetic elements) are a critical factor in the evolution, adaptation, pathogenicity and virulence of *S. aureus*, but it is well recognized that their presence/absence varies significantly among *S. aureus* strains and CCs [6,9,32]. Accessory genes can also move between lineages, which potentially changes lineage designations over time if accessory genes are used in the definition of lineages. Therefore, defining strains and lineages based upon core genes shared by all (or nearly all) strains of a species provides a stable measure of the population structure, and the analysis of accessory genes is complementary to the core gene analyses.

One of the challenges of proposing a new classification system for bacterial species with existing nomenclatures is achieving consistency with the currently accepted schema. The *S. aureus* cgMLST scheme and LIN code system proposed here mitigate this by having backwards compatibility with the major CCs, and the PubMLST website and BIGSdb platform provide the cgMLST and LIN code nomenclature for all users. This hierarchical LIN code classification threshold system delineates the *S. aureus* population structure and enables the user to choose the level of resolution they require. The designated LIN code ‘lineage’ is highly concordant with CC, and LIN code threshold 5 is largely concordant with ST, providing consistency across the available genotyping methods.

The *S. aureus* genome database was initially established by downloading and assembling sequencing data from the ENA, and relatively limited provenance data and phenotype data were available for each genome sequence. This has limited effects on the development of a robust genotyping scheme but does preclude a more detailed investigation into the epidemiology and evolution of defined genetic clusters. More recent submissions include more detailed and complete data, and we encourage future submitters of genome data to PubMLST to include as much provenance data as possible, for the benefit of the wider community. We also encourage the ongoing submission of *S. aureus* genome data from many geographical locations and with a wide range of epidemiological characteristics to PubMLST, to capture the full spectrum of genetic diversity within the *S. aureus* population. Finally, there have been many named clones, lineages and reference strains published over the last two decades: it may be time for an international *S. aureus* expert committee to be formed to ratify reference strains and genetic lineages and harmonize the naming of important *S. aureus*.

In conclusion, a high-resolution, open access cgMLST scheme and LIN code system is proposed, which enables the detailed analyses of genetic lineages within the *S. aureus* population. The varying levels of resolution provided by these new tools will be advantageous for *S. aureus* outbreak investigations, epidemiological surveillance and addressing research questions related to * S. aureus* evolution, pathogenesis and antimicrobial resistance.

## Supplementary material

10.1099/mgen.0.001486 Uncited Supplementary Material 1.

10.1099/mgen.0.001486 Uncited Supplementary Material 2.

## Abbreviations

AMR: antimicrobial resistance
ANI: average nucleotide identity
ARI: adjusted Rand Index
CCs: clonal complexes
cgMLST: core genome multilocus sequence typing
cgST: core genome sequence type
COG: Clusters of Orthologous Groups
ENA: European Nucleotide Archive
LIN: life identification number
MLST: multilocus sequence typing
MRSA: methicillin-resistant *Staphylococcus aureus*
rMLST: ribosomal multilocus sequence typing
rST: ribosomal sequence type
STs: sequence types
