# Supplementary material for: Development of a core genome multilocus sequence typing scheme and life identification number code classification system for Staphylococcus aureus
Source: Microb Genom. 2025 Aug 29;11(8):001486. doi: 10.1099/mgen.0.001486 (PMC12452198; doi:10.1099/mgen.0.001486)
Supplement: Uncited Supplementary Material 1. [file mgen-11-01486-s001.pdf]

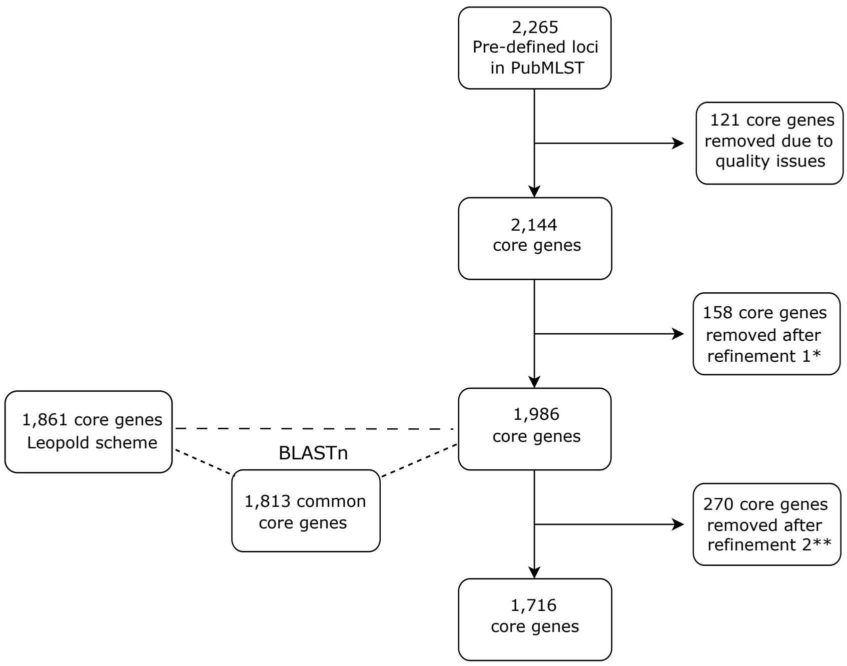

Refinements: removal of loci that had <95% (*) or <99% (**) allele assignments in the development dataset.

Supplementary Figure 1. Flowchart describing the selection of core gene loci included in the *S. aureus* cgMLST scheme. The Leopold et al scheme was published in J Clin Microbiol 2014 Jul;52(7):2365-70. doi: 10.1128/JCM.00262-14.

A

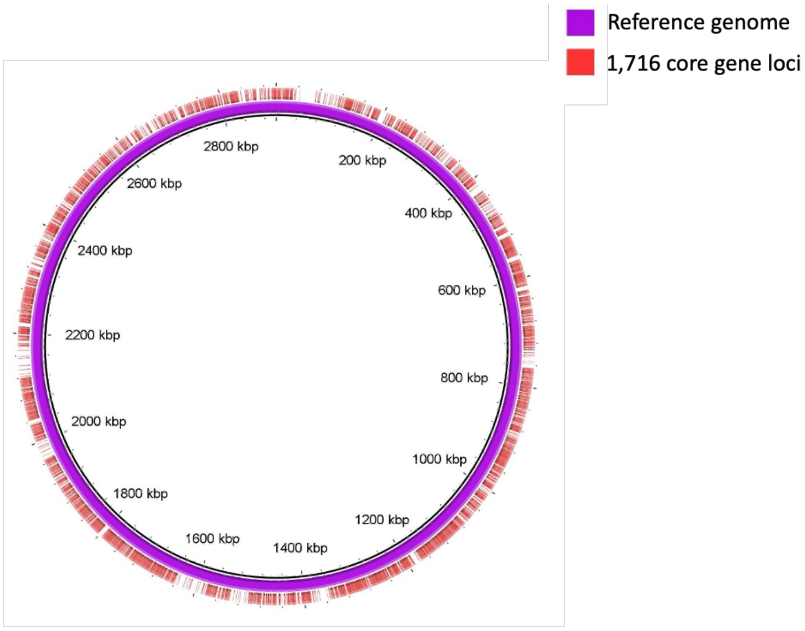

B

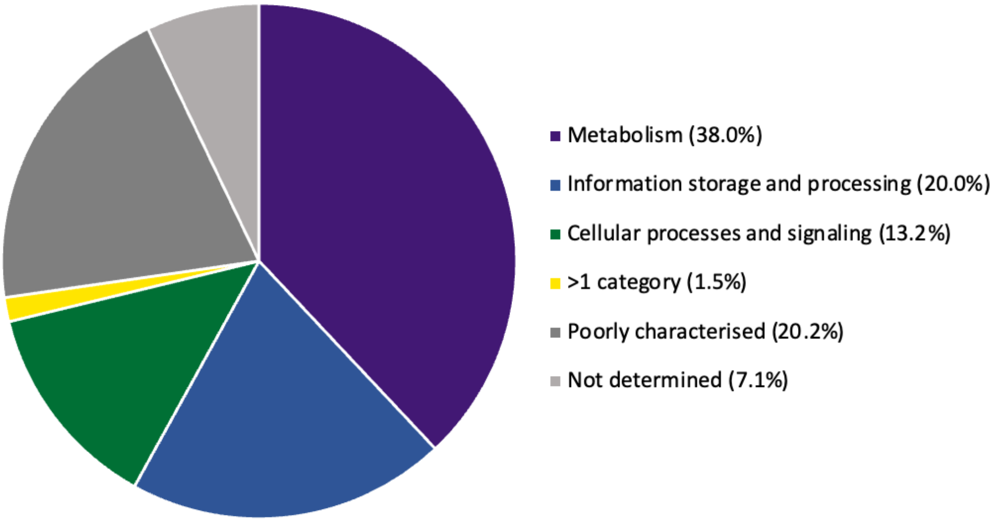

Supplementary Figure 2. Core gene loci included in the *S. aureus* cgMLST scheme. A. Distribution of the 1,716 core gene loci across the *S. aureus* reference genome MRSA252 (GenBank accession number BX571856). B. Predicted gene functions of the 1,716 core genes in the cgMLST scheme.

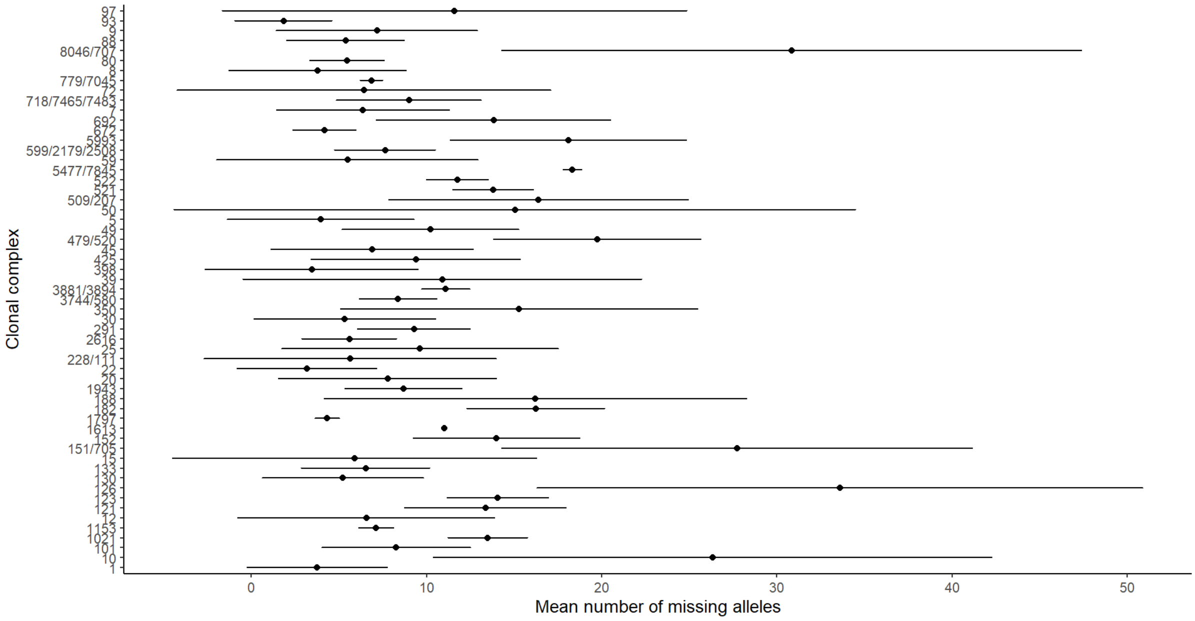

Supplementary Figure 3. Mean number of missing cgST alleles per genome for each of 57 clonal complexes with five or more members. Horizontal bars represent the standard deviation of the mean.

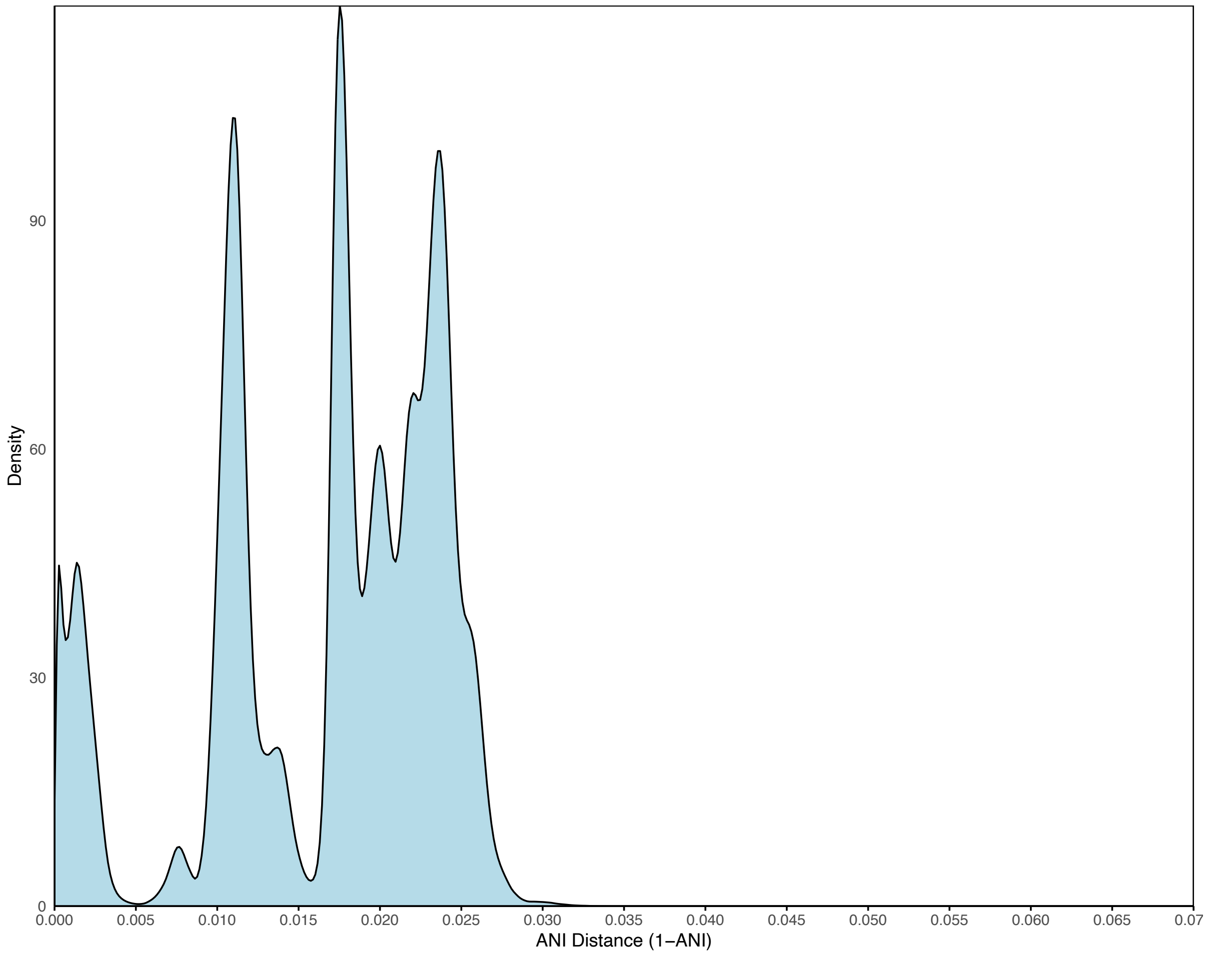

Supplementary Figure 4. Results of an ANI analysis of the development dataset of 5,000 *S. aureus* genomes.

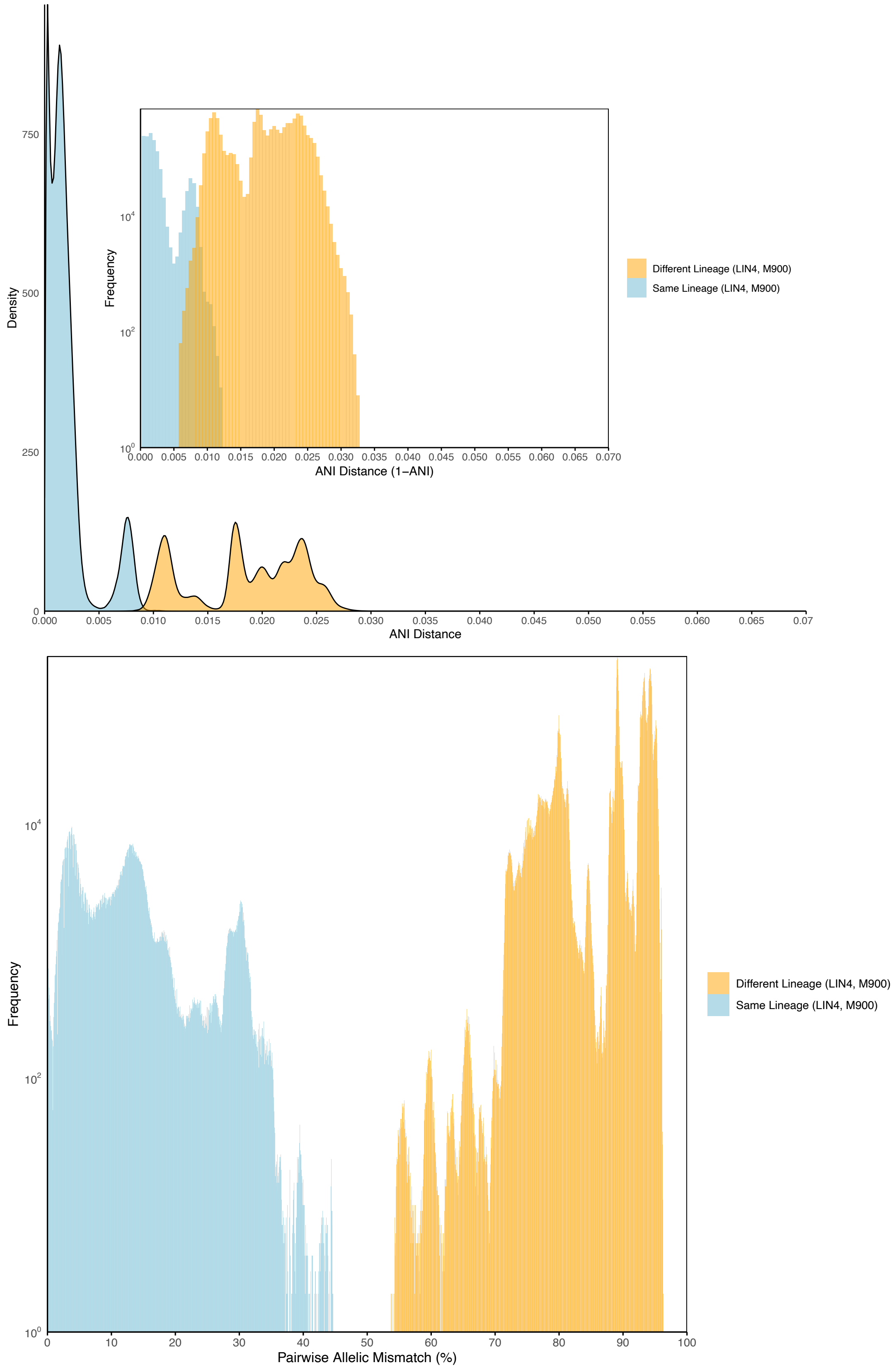

Supplementary Figure 5. Characterisation of *S. aureus* genomes by ANI distances and pairwise allelic mismatches, coloured by LIN code lineage (LIN 4 threshold, 900 pairwise mismatches). The Silhouette score computed using the ANI-based distance matrix for M900 was 0.82.

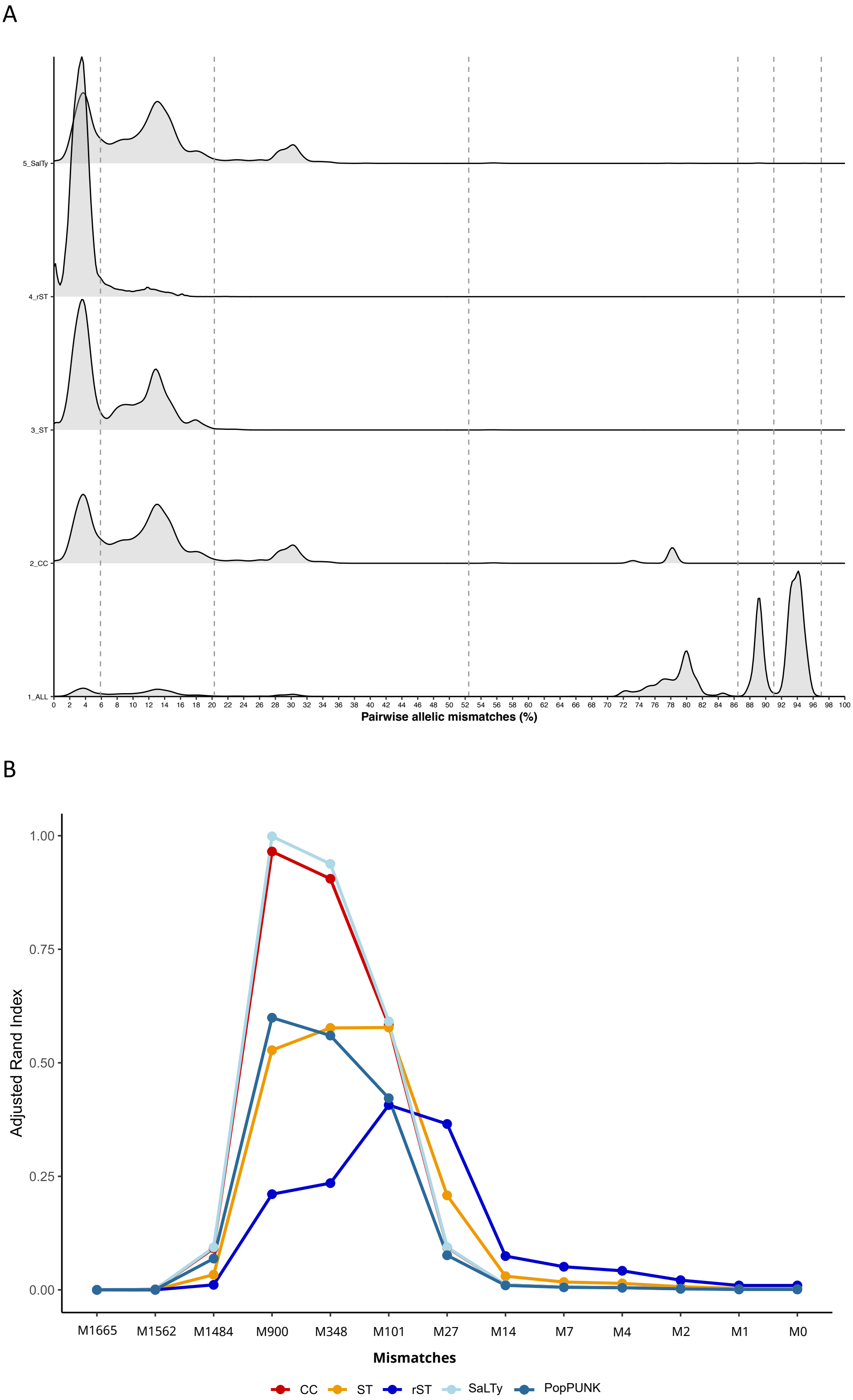

Supplementary Figure 6. Characterisation of the development dataset of 5,000 *S. aureus* genomes by cgMLST and comparison to other genotyping methods. Panel A depicts the density distributions of pairwise allelic differences between genomes belonging to a matching rST, ST, CC or SaLTy group. Panel B plots the concordance between clusters of *S aureus* at each of the 13 LIN code thresholds and the corresponding rST, ST, CC, SaLTy and PopPUNK designations. (Technical issues prevented a SaLTy analysis of the full genome dataset; ST and PopPUNK results were similar.)

A B C

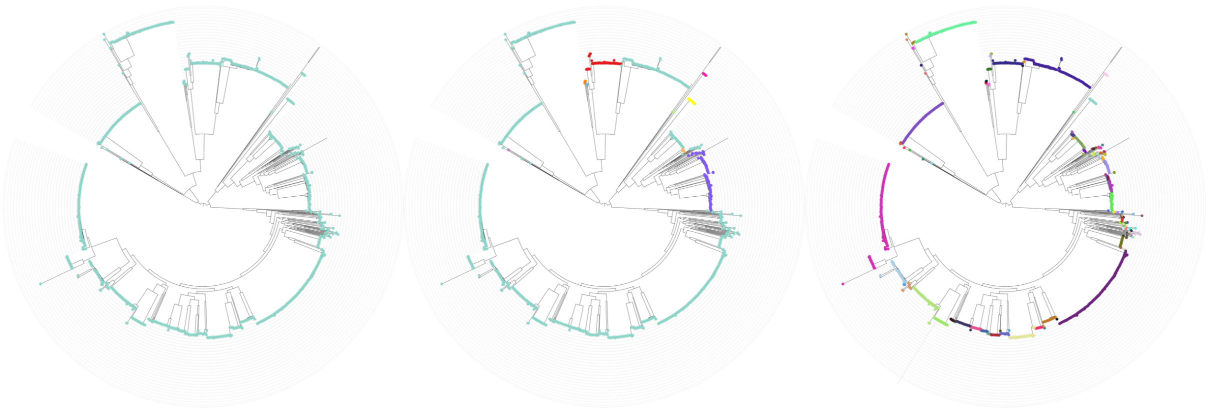

Supplementary Figure 7. Phylogenetic analysis of *S. aureus* genomes. The phylogenetic tree was constructed with a concatenated alignment of 1,716 core genome loci from one randomly selected genome representative of each of the 1,558 unique seven-locus sequence types. IQ-TREE was used with the GTR+G model of nucleotide substitution and 1,000 bootstrap replicates. The tree is rooted at the midpoint. The tree was annotated by LIN code groups at three classification levels: (A) LIN code classification threshold 1, (B) LIN code classification threshold 3, and (C) LIN code classification threshold 4 or 'lineage'.

Supplementary Table 1. List of all clonal complexes and singletons[a] in the full *S. aureus* study dataset.

CC	Genomes (n)	% of dataset
5	6405	24.0
8	5547	20.8
22	5107	19.1
398	1700	6.4
30	1514	5.7
1	903	3.4
45	683	2.6
130	549	2.1
15	530	2.0
93	469	1.8
97	384	1.4
133	313	1.2
9	277	1.0
59	243	0.9
188	211	0.8
425	167	0.6
88	143	0.5
121	134	0.5
25	126	0.5
72	105	0.4
12	97	0.4
7	91	0.3
151/705	90	0.3
228/111	66	0.2
49	59	0.2
20	57	0.2
39	48	0.2
101	42	0.2
1943	39	0.1
3881/3894	32	0.1
522	32	0.1
80	31	0.1
672	31	0.1
152	30	0.1
182	29	0.1
291	28	0.1
50	22	0.1

123	16	0.1
126	13	0.0
692	12	0.0
599/2179/2508	11	0.0
5993	9	0.0
1797	9	0.0
479/520	8	0.0
3744/580	8	0.0
1153	8	0.0
1021	8	0.0
718/7465/7483	8	0.0
779/7045	8	0.0
350	7	0.0
5477/7845	6	0.0
1613	6	0.0
10	6	0.0
8046/707	6	0.0
521	5	0.0
509/207	5	0.0
2616	5	0.0
3904	4	0.0
395/8123	4	0.0
3142	4	0.0
1290/6508	3	0.0
2328	3	0.0
96	3	0.0
816	3	0.0
3497	3	0.0
5360	3	0.0
2096/1760	2	0.0
7760	2	0.0
913	2	0.0
3837	2	0.0
1956	2	0.0
2990	2	0.0
1626	2	0.0
3111	2	0.0
7314	2	0.0
5365/7671	2	0.0
1027/5814	2	0.0
6063/6064	2	0.0
1292/1566	2	0.0

6869	2	0.0
3675/1094	2	0.0
1035	2	0.0
574	2	0.0
1073	2	0.0
1345	2	0.0
7047	2	0.0
5495/6624	2	0.0
5711	2	0.0
6610	2	0.0
7689/7691	2	0.0
1660	1	0.0
42	1	0.0
8083	1	0.0
3691	1	0.0
7747	1	0.0
3726	1	0.0
8048	1	0.0
3742	1	0.0
1725	1	0.0
1558	1	0.0
7694	1	0.0
136	1	0.0
7751	1	0.0
3752	1	0.0
7847	1	0.0
3834	1	0.0
8073	1	0.0
135	1	0.0
699	1	0.0
3846	1	0.0
3206	1	0.0
2098	1	0.0
7692	1	0.0
4968	1	0.0
7745	1	0.0
4976	1	0.0
7749	1	0.0
4981	1	0.0
7753	1	0.0
4984	1	0.0
7840	1	0.0

5032	1	0.0
7880	1	0.0
2099	1	0.0
8050	1	0.0
5474	1	0.0
8077	1	0.0
5491	1	0.0
2678	1	0.0
5494	1	0.0
709	1	0.0
2106	1	0.0
1727	1	0.0
5835	1	0.0
942	1	0.0
356	1	0.0
3689	1	0.0
6083	1	0.0
7693	1	0.0
2108	1	0.0
7695	1	0.0
6612	1	0.0
7746	1	0.0
6711	1	0.0
7748	1	0.0
6715	1	0.0
7750	1	0.0
6868	1	0.0
7752	1	0.0
2119	1	0.0
7754	1	0.0
2120	1	0.0
7799	1	0.0
2233	1	0.0
7841	1	0.0
6989	1	0.0
7849	1	0.0
407	1	0.0
8043	1	0.0
7110	1	0.0
8049	1	0.0
7258	1	0.0
8053	1	0.0

7306	1	0.0
8075	1	0.0
2442	1	0.0
8081	1	0.0
7471	1	0.0
2657	1	0.0
7479	1	0.0
2767	1	0.0
7494	1	0.0
2891	1	0.0
7497	1	0.0
2933	1	0.0
7499	1	0.0
890	1	0.0
7550	1	0.0
1766	1	0.0
7565	1	0.0
3432	1	0.0
7592	1	0.0
1768	1	0.0
7687	1	0.0
3646	1	0.0
7688	1	0.0
7690	1	0.0
6979	1	0.0
6984	1	0.0
3743	1	0.0
3747	1	0.0
Total	26,677	100

a. Singletons are unclustered STs of 1 genome each.

Supplementary Table 2. Results of Silhouette analyses using pairwise allelic mismatches and ANI distance.

Mismatches	St (pairwise allelic mismatches)	St (ANI distance)
1665	0	NA
1562	0.1609735	0.294619866
1484	0.23015173	0.24236917
900	0.82248053	0.816303894
348	0.72981808	0.755613185
101	0.3673041	0.004034104
27	0.21599369	-0.093228526
14	0.2474056	0.036517111
7	0.23148327	0.065570142
4	0.20373387	0.049378579
2	0.15691795	0.020801944
1	0.13299629	0.00243079
0	NA	0.002587129

Note: NA = not available
